# Herpesviruses and the Unfolded Protein Response

**DOI:** 10.3390/v12010017

**Published:** 2019-12-21

**Authors:** Benjamin P. Johnston, Craig McCormick

**Affiliations:** 1 Department of Microbiology & Immunology, Dalhousie University, 5850 College Street, Halifax, NS B3H 4R2, Canada; ben.johnston@dal.ca; 2 Beatrice Hunter Cancer Research Institute, 5850 College Street, Halifax, NS B3H 4R2, Canada

**Keywords:** unfolded protein response (UPR), integrated stress response (ISR), ATF6, IRE1, XBP1, PERK, ATF4, GADD34, herpesvirus, Kaposi’s sarcoma-associated herpesvirus (KSHV), cytomegalovirus (CMV), herpes simplex virus (HSV)

## Abstract

Herpesviruses usurp cellular stress responses to promote viral replication and avoid immune surveillance. The unfolded protein response (UPR) is a conserved stress response that is activated when the protein load in the ER exceeds folding capacity and misfolded proteins accumulate. The UPR aims to restore protein homeostasis through translational and transcriptional reprogramming; if homeostasis cannot be restored, the UPR switches from “helper” to “executioner”, triggering apoptosis. It is thought that the burst of herpesvirus glycoprotein synthesis during lytic replication causes ER stress, and that these viruses may have evolved mechanisms to manage UPR signaling to create an optimal niche for replication. The past decade has seen considerable progress in understanding how herpesviruses reprogram the UPR. Here we provide an overview of the molecular events of UPR activation, signaling and transcriptional outputs, and highlight key evidence that herpesviruses hijack the UPR to aid infection.

## 1. Overview of the Unfolded Protein Response

**Figure 1:**
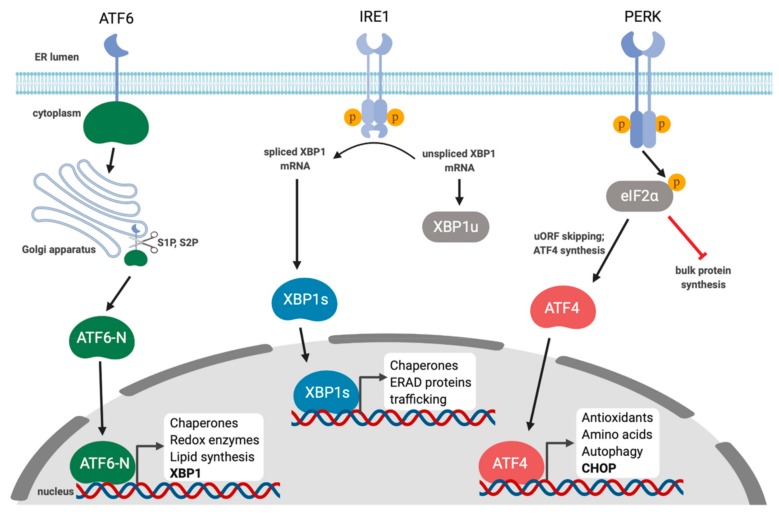
Endoplasmic reticulum (ER) stress activates the unfolded protein response. The accumulation of misfolded proteins in the ER activates the unfolded protein response (UPR). UPR sensor proteins ATF6, IRE1, and PERK are normally restrained by binding to the ER chaperone BiP; ER stress displaces BiP and activates the sensors, thereby promoting synthesis of UPR transcription factors that coordinate an ER stress-mitigating gene expression program. Specifically, in response to ER stress, ATF6 translocates to the Golgi and is proteolytically cleaved by site-1 protease (S1P) and site-2 protease (S2P), releasing the N-terminal cytoplasmic transcription factor ATF6-N. IRE1 is a kinase and endoribonuclease that splices out a 26-nucleotide intron on *XBP1* mRNA, which causes a translational frameshift to generate the transcription factor XBP1s. PERK phosphorylates eIF2α, which attenuates bulk translation thereby reducing protein load in the ER. Increased eIF2α phosphorylation also causes the selective translation of the transcription factor ATF4. ATF6-N, XBP1s, and ATF4 transactivate genes involved in protein folding, degradation of misfolded proteins, lipid synthesis, and antioxidant responses.

The endoplasmic reticulum (ER) coordinates diverse cellular functions including lipid synthesis, calcium storage, and protein synthesis [1]. The ER is the gateway to the secretory pathway and one-third of the proteome is translated in the ER. These newly translated proteins need to be folded, modified, and sorted. The ER proteostasis (a portmanteau of protein and homeostasis) network prevents protein misfolding by matching protein load to ER folding capacity [1]. This is largely achieved through a network of enzymes that promote folding and degrade terminally misfolded or aggregated proteins through ER-associated degradation (ERAD) and autophagy. However, dramatic changes in physiology or exposure to environmental stressors can shift this balance, resulting in accumulation of misfolded proteins, a process known as ER stress. All eukaryotic cells have evolved mechanisms to sense and respond to ER stress; when stress is detected, cells attempt to restore proteostasis by increasing expression of genes that regulate ER protein folding and degradation. This conserved response to ER stress is known as the unfolded protein response (UPR) [2]. In metazoans, the UPR is initiated by three ER-localized integral membrane proteins, PKR-like endoplasmic reticulum kinase (PERK), Activating Transcription Factor 6 (ATF6), and Inositol-Requiring Enzyme 1 (IRE1), that are normally maintained in an inactive state by the abundant ER chaperone Immunoglobulin Heavy Chain-Binding Protein (BiP; also known as Glucose-Regulated Protein, 78 kDa, Grp78) (Figure 1). During ER stress, BiP dissociates from the luminal domains of UPR sensors and binds exposed hydrophobic surfaces on unfolded proteins; BiP displacement triggers UPR sensor activation [3,4]. These sensors work in concert to restore ER protein homeostasis through transcriptional and translational reprogramming. This is accomplished by transiently attenuating bulk protein translation, increasing chaperone and foldase synthesis, expanding ER surface area by increasing phospholipid synthesis, and degrading terminally misfolded proteins. Persistent or irremediable ER stress causes the UPR to transition from an adaptive response to a pro-apoptotic response to prevent further damage to the host [2].

Since the discovery of IRE1, PERK, and ATF6 as the three sensors of the UPR, there have been many studies investigating whether viruses affect ER stress and the UPR. The UPR was first characterized for its ability to upregulate two highly abundant ER chaperones, BiP and Grp94, and two early studies showed that infection with Simian virus 5 or introduction of a mutant form of influenza A virus hemagglutinin (HA) protein into cells caused accumulation of these chaperones [5,6]. Our understanding of the interplay between herpesviruses and the UPR remains incomplete, but studies to date indicate a complex mode of regulation. This review will provide an overview of the molecular signaling events of the UPR and broader impacts on human health and provide examples of how different herpesviruses usurp the UPR to create an optimal niche for replication.

## 2. IRE1 Is a Kinase and an Endoribonuclease

IRE1 is a type I ER-resident transmembrane protein that contains an ER stress-sensing amino-terminal luminal domain and cytoplasmic carboxy-terminal protein kinase and endoribonuclease domains [7]. IRE1 has two isoforms in mammals, IRE1α and IRE1β. IRE1α is expressed in all tissues, whereas IRE1β expression is primarily restricted to bronchial and intestinal epithelial cells [8]. IRE1 is maintained as an inactive monomer through stable binding of BiP to its luminal domain. With the onset of ER stress, BiP is displaced from IRE1 due to its higher affinity for misfolded proteins, which allows dimerization of IRE1 luminal domains and trans-autophosphorylation [9]. Subsequent assembly of higher-order oligomers drives IRE1 activity [10]. IRE1 phosphorylation facilitates ATP binding, which induces a conformational change that activates the C-terminal ribonuclease (RNase) domain [11]. The activated RNase targets the *XBP1* mRNA for cleavage, which removes a short intron (26-nucleotides in mammalian cells) that is re-ligated by the tRNA ligase RTCB [12,13,14,15,16]. Splicing shifts the downstream open reading frame and generates XBP1-spliced (XBP1s), a protein with an extended carboxy-terminus that fuses a transcription activation domain onto the basic leucine zipper (bZIP) domain, yielding a functional transcription factor. XBP1s translocates to the nucleus and binds to consensus promoter elements that contain a core ACGT motif, found in genes that encode proteins involved in protein folding, ER-associated degradation (ERAD) and lipid biosynthesis [17,18].

The *XBP1* mRNA is the sole known substrate for IRE1-mediated splicing, with two adjacent stem-loops with IRE1 RNase cleavage sites that are positioned to enable RTCB-mediated ligation [11]. Other ER-targeted mRNAs with single XBP1-like stem-loops can be cleaved by IRE1 and subsequently degraded by the 5′–3′ exonuclease XRN1 through a process known as regulated IRE1-dependent decay (RIDD) [19,20,21]. The differential regulation of these two IRE1 functions remains very poorly understood, but there is evidence for inverse correlation. For example, RIDD is increased in *XBP1*-deficient cells [21]. IRE1 kinase activity is required for RIDD; bypassing IRE1 kinase activity by using an IRE1 point mutant that can bind the non-hydrolysable ATP analog, 1NM-PP1, prevents RIDD but permits *XBP1* splicing [19]. In vitro studies suggest that IRE1 levels and oligomeric state influence functional outcomes, whereby low IRE1 levels favor dimer formation and RIDD, and high IRE1 levels promote the formation of higher-order oligomers that favor *XBP1* splicing [22]. However, this observation has yet to be validated in intact living cells.

## 3. PERK and the Integrated Stress Response

In response to stress, eukaryotic cells attenuate global synthesis of non-essential proteins. Protein synthesis is governed by the activity of the heterotrimeric eIF2 (eukaryotic initiation factor 2) GTPase that binds methionine-loaded tRNAi^Met^. eIF2 is a heterotrimer of eIF2α, β, and γ, where the γ subunit directly binds GTP and Met-tRNAi^Met^ [23]. The α and β subunits stabilize the interaction with Met-tRNAi^Met^, and as discussed below, the α subunit has regulatory activity [24]. When bound to GTP, eIF2 binds Met-tRNAi^Met^ to form the ternary complex (TC). The TC is loaded onto the 40S small ribosomal subunit, and subsequent recruitment of eIF1, eIF1A, eIF3, and eIF5 drives formation of the 43S pre-initiation complex (PIC). The 43S PIC indirectly binds to the 5′-cap of mRNAs by binding the eIF4F heterotrimeric complex (composed of eIF4E, eIF4G, and eIF4A). eIF4F links the 5′-cap to the poly(A) tail by binding poly(A)-binding protein (PABP). The 43S PIC initiates scanning of the 5′UTR in a 5′–3′ direction to find the AUG start codon. Once Met-tRNAi^Met^ engages with the start codon GTP is hydrolyzed to GDP and eIF2 is released. Released eIF2 can participate in a new translation initiation cycle, but this is contingent upon exchange of GDP-bound eIF2 for GTP by the guanine nucleotide exchange factor (GEF) eIF2B [23].

In response to different types of stress, the α subunit of eIF2 can be phosphorylated on Serine 51 by stress-specific eIF2α kinases, which induces higher affinity binding to eIF2B, inhibiting GEF activity and thereby reducing translation. Mammals encode four different eIF2α kinases that are activated by different types of stress: GCN2 (general control nonderepressible 2), PERK, PKR (Protein kinase R; “R” stands for RNA) and HRI (heme regulated inhibitor) [25,26]. Through these eIF2α kinases, the cell can sense diverse stresses and respond by attenuating global translation through eIF2α phosphorylation [27,28]. This initiates a cellular stress response known as the integrated stress response (ISR) [29].

Like IRE1, PERK dimerizes in response to ER stress and undergoes trans-autophosphorylation [25,26]. This induces a conformational change that allows PERK to bind and phosphorylate the translation initiation factor, eIF2α [30,31]. The PERK and IRE1 luminal domains are quite similar, which highlights a conserved mechanism of responding to ER stress via disengagement of BiP [3,31,32]. A central feature of the ISR is the phosphorylation of eIF2α to attenuate bulk translation, while also specifically increasing production of stress-related proteins due to the presence of uORFs in the 5′-UTR of their mRNAs [33,34]. In the absence of stress, the 43S PIC initiates protein synthesis on these uORFs; eIF2α phosphorylation alters the stringency of scanning by the 43S PIC, causing uORF bypass and downstream translation initiation on longer ORFs that encode stress-mitigating proteins like the bZIP transcription factor Activating Transcription Factor 4 (ATF4). ATF4 transactivates genes involved in amino acid biosynthesis, the anti-oxidant response and autophagy [29]. It is a member of the ATF/CREB family of transcription factors and can bind to ATF/CRE-like sequences with a consensus sequence of 5′-TGACGTCA-3′, and amino acid response elements (AAREs) that have a conserved core sequence 5′-ATTGCATCA-3′ [35]. ATF4 amplifies ISR transcriptional responses by transactivating genes encoding the bZIP transcription factors Activating Transcription Factor 3 (ATF3) and C/EBP-Homologous Protein (CHOP) [33,36]. ATF4 can also heterodimerize with CHOP and change the repertoire of transactivated genes [25,37,38]. Recently, a small molecule called ISRIB (ISR inhibitor) was shown to bind eIF2B and promote GEF activity, which attenuated translation inhibition following activation of an eIF2α kinase, thereby suppressing ATF4 and CHOP synthesis [39,40,41,42]. CHOP transactivates *Growth Arrest and DNA-Damage-Inducible 34 (GADD34)* [43]; GADD34 recruits protein phosphatase 1α (PP1α) to phospho-eIF2α to enforce eIF2α dephosphorylation and restore bulk translation [44]. However, these GADD34-PP1α phosphatase complexes operate irrespective of stress resolution, which can impact cell fate (discussed below). Like ATF4, both CHOP and GADD34 mRNAs contain uORFs that are required for maximal translation following engagement of the ISR [45,46,47].

## 4. ATF6 Is Activated by Regulated Intramembrane Proteolysis

Another sensor of ER stress is ATF6, a 90 kDa ER-localized type II transmembrane glycoprotein with two isoforms known as ATF6α and ATF6β [48,49,50]. Both isoforms are expressed in all tissue types, and may have some redundant roles, but ATF6α predominates in UPR signaling in response to ER stress [51]. Similar to IRE1 and PERK, in the absence of stress BiP binds ATF6 to inhibit signaling [52]. There are likely other modes of maintaining ATF6 in an inactive state, including the formation of intra- and inter-molecular disulfide bonds with other ATF6 proteins, creating ATF6 dimers and oligomers [53]. In response to ER stress, the disulfide bonds are reduced and BiP is released, which allows ATF6 to translocate to the Golgi in a COPII-dependent manner where it is cleaved at the luminal side of the ER by Site-1 protease (S1P) and the cytoplasmic side of the ER by Site-2 protease (S2P) in a process referred to as regulated intramembrane proteolysis (RIP) [54,55]. These cleavage events release the N-terminal cytosolic fragment, ATF6-N, which is an active bZIP transcription factor that traffics to the nucleus and transactivates genes to mitigate ER stress.

ATF6-N homodimers bind to conserved consensus motif called ERSE (for ER stress response element; consensus sequence CCAAT-N9-CCACG) and ERSE-II (consensus sequence ATTGG-N-CCACG), which are found in UPR genes that encode chaperones, quality control proteins and ERAD proteins, as well as redox pathway components [56,57,58]. Two of the best-characterized ATF6 target genes are the abundant ER chaperones BiP and Grp94 [57,59,60,61]. ATF6 also interacts with the heterotrimeric transcription factor NF-Y (composed of NF-YA, NF-YB, and NF-YC) and this interaction is required for ATF6 binding to ERSE [58]; in this complex, ATF6 dimers bind to CCACG and NF-Y binds CCAAT [62]. ATF6 transactivates the ISR gene CHOP via an ERSE in the CHOP promoter [32,62]. ATF6 has also been shown to transactivate *XBP1* [13] and heterodimerize with XBP1s to transactivate a distinct set of UPR genes [51,57].

The mechanism of how ATF6 trafficking and cleavage is regulated following ER stress is not well understood. It was found that only the luminal domain of ATF6 is involved in ER stress sensing and trafficking to the Golgi; swapping the carboxy-terminal luminal domain of ATF6 onto the constitutively Golgi-transported protein Sec22 caused the hybrid protein to be retained in the ER until it could be released to the Golgi in response to ER stress [63]. COPII is located on the cytoplasmic surface of the ER and therefore it is not known how the luminal domain of ATF6 signals to COPII for trafficking [54,64]. This mechanism of transcription factor synthesis via ER-to-Golgi trafficking and proteolysis of a precursor protein is not unique to ATF6; Sterol Regulatory Element Binding Transcription Factor 1 (SREBP1) and SREBP2 are ER-localized proteins that relocate to the Golgi when cholesterol is scarce and undergo RIP by S1P and S2P that releases active transcription factors that transactivate genes involved in cholesterol biosynthesis. The SREBPs detect cholesterol scarcity by interacting with SCAP (SREBP cleavage activating protein). SREBP and SCAP are retained in the ER by INSIG (insulin-induced gene) binding of SCAP; low sterol levels cause release of INSIG from SCAP, allowing SCAP and SREBP to translocate to the Golgi in COPII vesicles [65]. By contrast, ATF6 processing does not require SCAP [55] and the molecular events that control ATF6 release to the Golgi remain elusive.

Additional ER transmembrane proteins that resemble ATF6 belong to the OASIS subfamily of bZIP transcription factors that include Luman/LZIP/CREB3, OASIS/CREB3L1, BBF2H7/CREB3L2, CREBH/CREB3L3 and CREB4/AIbZIP/Tisp40/CREB3L4 [66]. Like ATF6 and SREBP1/2, these proteins are also processed by RIP to release amino-terminal bZIP transcription factors. Other than Luman, these proteins have cell- or tissue-specific modes of expression that suggest distinct physiological roles. Some of these ATF6-like proteins can also be activated by ER stress, suggesting that the UPR may have additional branches that operate in different tissues [66].

## 5. Crosstalk among the Branches of the UPR

Although activation of the three UPR sensors during ER stress elicits distinct transcriptional outputs, there is accumulating evidence of coordination and co-regulation between the different branches. Furthermore, the type and duration of stress seems to impact the output of each branch of the UPR. For example, Peter Walter’s group showed that lethal doses of ER stress-inducing molecules thapsigargin or tunicamycin elicit sustained, long-term PERK activity, but only transient IRE1 and ATF6 activity [67]. Thus, it appears that UPR resolution mechanisms are quite asynchronous between the different branches of the pathway.

The attenuation of prolonged IRE1 signaling is not fully understood but a recent study showed that the phosphatase RNA Polymerase II Associated Protein 2 (RPAP2) is activated in a PERK-dependent manner and dephosphorylates IRE1 [68]. PERK silences *XBP1* expression by upregulating microRNA 30c-2-3p, which binds and diminishes translation of the *XBP1* mRNA; this may serve as another mechanism of attenuating IRE1 signaling after prolonged ER stress [69]. Moreover, XBP1s transactivates the gene encoding hsp40 co-chaperone ERdj4, which binds to IRE1 and facilitates BiP recruitment and stabilization of inactive IRE1 monomers [70]. Thus, it seems that IRE1 signaling can be attenuated via multiple independent mechanisms that facilitate a return to homeostasis.

XBP1s may also negatively regulate PERK activity by increasing production of another hsp40 family member, p58^IPK^ (also known as DNAJC3) [71]. p58^IPK^ was originally identified as a PKR inhibitor [72], but since PERK and PKR share sequence similarity in the cytoplasmic domain, it is not surprising that p58^IPK^ also represses PERK. As a co-chaperone for BiP, p58^IPK^ may inhibit PERK via BiP recruitment [73]. Thus, sustained IRE1 activity and XBP1s accumulation may inactivate PERK through p58^IPK^ upregulation, although this has not been demonstrated experimentally.

There is evidence that PERK activation can suppress IRE1 signaling, but there has also been a report that demonstrated that the ISR can promote IRE1–XBP1 signaling via ATF4 transactivation of *IRE1* [74] or increased stability of spliced *XBP1* mRNA [75]. These conflicting observations may be due to differences in cell models and environmental conditions in these studies. Therefore, further analysis is required to clarify how PERK affects IRE1 activity.

As previously mentioned, by transactivating *XBP1* and *CHOP* genes, ATF6 controls the output of the IRE1 and PERK branches of the UPR [13,62]. ATF6/XBP1s heterodimeric complexes enhance and broaden UPR transcriptional outputs compared to XBP1 and ATF6 homodimers [57]. ATF6 plays a lead role in transactivating the *BiP* gene, but ATF4, with the help of ATF1 and CREB1, has also been reported to transactivate *BiP* [76]. ISR activation promotes translation of *BiP* mRNAs via uORF skipping [77], but this mechanism involves eIF2A rather than eIF2α; eIF2A is a functional homolog of eIF2 that does not rely on eIF2B for GTP cycling, and promotes translation initiation on non-canonical CUG start codons [78]. Together, these examples demonstrate widespread crosstalk between UPR branches that promote robust and tightly coordinated responses to ER stress.

## 6. The UPR and Cell Fate

Sustained, unresolved ER stress causes the UPR to switch from an adaptive response to a pro-apoptotic response [2]. This switch from adaptation to apoptosis depends on the strength, type, and duration of stress. The precise mechanisms that govern this switch are not well understood. Furthermore, some of the pro-apoptotic factors induced by the UPR, such as CHOP, are induced during the adaptive phase and it is not clear how these pro-apoptotic factors are regulated to ensure that their apoptotic functions are implemented only after the cell has reached a “tipping point” after which the stress cannot be resolved [79]. Nonetheless, there have been considerable efforts to elucidate mechanisms of UPR-mediated apoptosis and identify factors that govern the switch from adaptive responses to apoptosis. Generally, it is thought that ATF6 plays a lead role in proteostasis, whereas IRE1 and PERK heavily influence cell fate [80]. However, confidence in these assignments is undermined by the intertwined nature of UPR regulation.

Sustained IRE1 signaling elicits apoptosis through multiple mechanisms including the mitogen-activated protein kinase (MAPK)-c-Jun N-terminal kinase (JNK) pathway. IRE1 can bind TRAF2 (TNF Receptor Associated Factor 2) and recruit and activate the MAP3K (MAP kinase kinase kinase) apoptosis signal-regulating kinase 1 (ASK1) [81,82]. ASK1 phosphorylates the MAPK JNK, which promotes apoptosis via inhibitory phosphorylation of the anti-apoptotic Bcl-2 protein, and activating phosphorylation of the pro-apoptotic Bim protein [83,84]. IRE1 can also promote apoptosis via RIDD. RIDD cleaves a variety of microRNAs, including miR-17, -34a, -96, and -125, all of which target pro-caspase-2; thus, RIDD-mediated destruction of key miRNAs causes accumulation of pro-caspase-2 [85]. This larger available pool of caspase-2 increases the capacity for cleavage of BID into active tBID, which activates the pro-apoptotic Bax protein [86]. However, RIDD does not immediately induce apoptosis in response to ER stress; in the early stages of the UPR, RIDD circumvents the extrinsic apoptosis pathway by cleaving the mRNA encoding Death Receptor 5 (DR5) and preventing caspase-8 activation [87]. Recently, the role of DR5 and caspase-8 activation in ER stress-induced apoptosis has been disputed [88]. Therefore, further work is needed to elucidate precise mechanisms that regulate IRE1-dependent cell death mechanisms in response to chronic ER stress.

PERK-mediated eIF2α phosphorylation arrests global protein synthesis while enabling selective translation of uORF-containing mRNAs encoding ATF4 and CHOP. CHOP is a pro-apoptotic transcription factor that transactivates *DR5* to promote apoptosis by activating caspase-8 [89]. CHOP also heterodimerizes with C/EBPα to transactivate *Bim* [84]; in turn, Bim activates Bax and disrupts mitochondrial function. CHOP further enforces mitochondrial dysfunction by repressing the anti-apoptotic *Bcl-2* gene [83]. CHOP also induces apoptosis in response to sustained ER stress through the upregulation of Endoplasmic Reticulum Oxidoreductase 1 alpha (Ero1α), which causes hyper-oxidation of ER resident proteins and additional protein misfolding [43]. Ero1α also activates the inositol-1,4,5-triphosphate (IP3) receptor (IP3R) leading to calcium release from the ER [90]. Large increases in cytoplasmic calcium can be taken up by the mitochondria, which has been shown to induce mitochondrial permeabilization and cytochrome c release [91]. ER calcium is required for protein folding and therefore secretion of calcium through the IP3R may also potentiate protein misfolding, leading to further toxicity [92].

As previously mentioned, CHOP transactivates *GADD34*, which directs dephosphorylation of eIF2α by PP1a, thereby restoring translation [43,44]. This is an important step during the adaptive phase of the UPR that accompanies stress resolution. However, apoptosis can be promoted by inappropriate resumption of global protein synthesis and translocation of nascent proteins into an ER that is already burdened by misfolded proteins [43]. GADD34 expression likely also facilitates translation of pro-apoptotic proteins [93]. It has also been shown that the combined action of ATF4 and CHOP, through both homo- and hetero-dimerization, transcriptionally upregulates genes that promote protein synthesis, including GADD34, and it is a combination of this increase in protein synthesis and oxidative stress that drives apoptosis [38]. Blocking the resumption of translation by inhibiting GADD34 during chronic ER stress can protect cells from apoptosis [94]. ATF4 and CHOP are upregulated during the adaptive phase of the UPR and therefore it is not entirely clear why early induction of these transcription factors does not pre-destine the cell to apoptosis. ATF4 proteins are quite labile, as are the mRNAs that encode them, so their levels should rapidly decline following stress resolution. However, sustained PERK activity allows these proteins to accumulate to promote apoptosis [79].

Much of what we know of the UPR signaling that controls cell fate is from the use of ER stress-inducing drugs that have gross deleterious effects on the ER and the cell. These include tunicamycin, which inhibits N-linked glycosylation; thapsigargin, which depletes ER calcium by inhibiting the SERCA (sarco/endoplasmic reticulum Ca^2+^-ATPase) pump; and reducing agents dithiothreitol (DTT) and β-mercaptoethanol that disrupt disulfide bonds. Therefore, many of these studies describing how the UPR controls the switch from restoring protein homeostasis may not be fully reflective of what is happening in vivo following a disruption in ER protein homeostasis. Developing better models, both in vitro and in vivo, that recapitulate physiologic ER stresses will help us better understand the precise contributions of the different UPR sensors in regulating apoptosis.

## 7. The UPR in Health and Disease

The UPR governs a variety of physiological processes and associated disease states [95]. Certain cells require the UPR and an augmented ER to meet increased demands on the secretory system during differentiation; these include plasma cells [96], pancreatic β cells [97], and granulocytic eosinophils [98]. In the intestinal epithelium, UPR signaling is required for differentiation and maintenance of cells with strong secretory phenotypes that guard against infection, including Paneth cells that secrete antimicrobial peptides and goblet cells that secrete large amounts of mucins to prevent pathogen infiltration past the intestinal barrier. Accordingly, knocking out *Xbp1* in mouse intestines eliminated Paneth cells and decreased levels of goblet cells, leaving them more susceptible to spontaneous enteritis and *Listeria monocytogenes* infection [99]. Paneth cell dysfunction also contributes to inflammatory bowel disease (IBD), including Crohn’s disease or ulcerative colitis [100]. *Xbp1* deletion in Paneth cells leads to increased ER stress, which can be mitigated by increased protein catabolism through autophagy [99].

While the UPR and specifically the transcription factor XBP1s is essential for plasma cell differentiation [96,101,102], dysregulated XBP1s signaling can also promote disease progression as overexpression of XBP1s can promote a plasma cell cancer known as multiple myeloma [103]. XBP1 and the UPR are implicated in other cancers, such as breast cancer [104] and ovarian cancer [105], as well as protein misfolding neurodegenerative disorders like Huntington’s disease [106]. Due to the importance of the UPR in insulin secretion by pancreatic β cells, dysregulated UPR signaling is also linked to type II diabetes [107].

The IRE1-XBP1 pathway is also important for the development and survival of many different immune cells, including dendritic cells (DCs) [108]. Depending on DC type or location, *XBP1* can be essential for survival or impact cell function, including surface presentation of class I major histocompatibility complex (MHC-I). Constitutive expression of XBP1s can promote lipid accumulation in DCs, which can inhibit antigen presentation [109,110]. IRE1-XBP1 signaling can be activated in CD8+ T cells in response to infection and *XBP1* loss abrogates effector T cell differentiation [111].

The IRE1-XBP1 pathway also likely plays an important role in regulating inflammation in response to infection. Activation of TLR2 or TLR4 in macrophages specifically engaged the IRE1-XBP1 pathway and XBP1 was required to augment expression of pro-inflammatory cytokines IL-6, TNFα, and IFN-β [112]. Engagement of TLR2 and TLR4 was reported to promote IRE1 polyubiquitination by TRAF6, which blocked IRE1 dephosphorylation by PP2A to prolong IRE1 signaling [113]. TLR signaling specifically activated the IRE1 branch of the UPR [112]. PERK and the ISR were also inhibited by TLR signaling following treatment with tunicamycin [114]. TLR signaling inhibited the ISR by PP2A-mediated dephosphorylation of the epsilon subunit of eIF2B [115]. Our understanding of the role for the UPR in immunity and inflammation is only in its infancy and employing different in vivo models for studying inflammation or using new technologies like single cell sequencing will surely help advance this field.

There is also accumulating evidence for UPR subversion during virus infection. Naturally, most studies to date have focused on enveloped viruses, since the synthesis of envelope glycoproteins in the ER can burden ER folding machinery to the point where it activates the UPR. Certain viruses have been shown to activate all three UPR branches, whereas other viruses appear to selectively activate or inhibit one of the branches. It is plausible that certain features of the UPR could aid viral replication. For example, UPR activation in response to infection stress could promote efficient viral protein synthesis and cell survival through increased synthesis of chaperone proteins and ER expansion. However, other aspects of the UPR could hinder viral replication by attenuating bulk translation and increasing degradation of viral proteins via ERAD. UPR-mediated apoptosis could limit replication of certain viruses, but others may benefit from apoptosis induction to aid dissemination of progeny [116]. The UPR can also impact broader responses to viral infection like autophagy and inflammation [117]. Because the UPR can exert complex virus-specific and cell type-specific effects on viral replication, it makes sense that certain viruses appear to “fine-tune” the UPR by selectively blocking one or more branches to aid viral replication. For the most part, we lack a mechanistic understanding of how the UPR impacts viral replication, or how viruses usurp the UPR to create an optimal environment for productive viral replication. However, important advances have been made through the study of herpesviruses over the past decade. Here, we will review the current understanding of herpesvirus interactions with the UPR, and viral mechanisms for UPR subversion.

## 8. Gammaherpesviruses and the UPR

Herpesviruses establish life-long persistent infection through latency, a quiescent state wherein viral gene expression is restricted to a handful of genes and the viral genome is usually maintained as a circular episome associated with host chromatin [118]. During mitosis, the viral episome is replicated by the cellular DNA polymerase and segregated to daughter cells. An essential feature of latency is reversibility; host signal transduction causes episome decondensation and initiates the full lytic viral gene expression program, which proceeds in an ordered, temporal cascade. The lytic cycle also features replication of the viral genome by a viral DNA polymerase, yielding a linear DNA product that is packaged into capsids to generate infectious progeny virions that can spread to new hosts.

**Figure 2:**
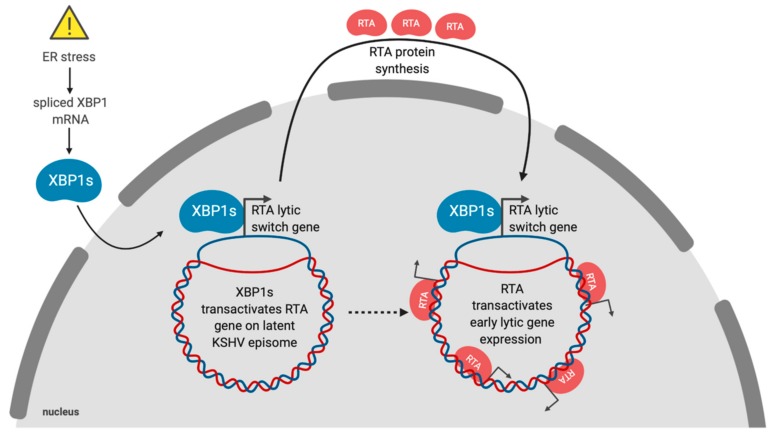
Kaposi’s sarcoma-associated herpesvirus (KSHV) reactivates from latency in response to ER stress. In response to ER stress, *XBP1* mRNA is spliced and the XBP1s protein accumulates and translocates to the nucleus, where it transactivates the *RTA* gene encoding the lytic switch protein. RTA binds and transactivates viral early gene promoters throughout the genome and commits the virus to the lytic gene expression program and virion production.

Latency is the default replication program for gammaherpesviruses, which include the human herpesviruses Epstein–Barr virus (EBV) and Kaposi’s sarcoma-associated herpesvirus (KSHV) [118]. The physiologic signals that control the switch from latency to lytic replication are unclear, but in cell culture viral reactivation has often been linked to exposure to stress-inducing stimuli. ER stress can trigger reactivation of EBV, KSHV and murine gammaherpesvirus 68 (MHV68) [119,120,121,122]. Lytic reactivation in response to ER stress is primarily due to XBP1s (Figure 2). The ER stress-sensing mechanism involves the presence of XBP1s target sequences in the promoters of immediate early viral genes. KSHV and MHV68 express the immediate early protein replication and transcriptional activator (RTA), which is essential and sufficient to induce lytic replication [123,124]. The RTA promoter in KSHV contains at least one XBP1s response element with an ACGT core motif [125]. The RTA promoter also contains hypoxia-inducible factor 1 (HIF-1) response elements, which also contain ACGT core sequences that enable KSHV reactivation in response to hypoxia [126]. Hypoxia was also shown to induce *XBP1* splicing, and robust RTA expression was dependent on both HIF-1 and XBP1s [125].

EBV lytic replication requires the immediate early proteins BRLF1 and BZLF1. While ER stress has been shown to trigger EBV reactivation from latency, XBP1s only minimally induces BRLF1 and BZLF1 expression, and robust expression requires simultaneous protein kinase D activation [119]. ER stress also induces the EBV oncoprotein Latent Membrane Protein 1 (LMP1) through direct transactivation by XBP1s and possibly via ATF4 as well [127]. The UPR can drive LMP1 expression, and newly synthesized LMP1 can sustain UPR activity, which may provide a feed-forward mechanism to further increase LMP1 production during EBV latency [128]. Thus, the UPR is tightly linked to EBV oncogenesis by inducing the expression of oncoproteins and controlling the reactivation and spread of the virus.

KSHV is a lymphotropic virus that infects primary B cells and causes their aberrant differentiation into proliferating primary effusion lymphoma (PEL) cells that resemble a form of pre-plasma cell (or plasmablast) [129,130,131]. As previously mentioned, XBP1s is essential for differentiation of B cells into non-dividing plasma cells (PCs) [96]. Normally, plasma cells develop in response to B cell receptor (BCR) activation following antigen recognition. In latently KSHV infected B cells, BCR activation can trigger XBP1s-dependent lytic reactivation [132]. Since PEL cells have a plasmablast-like phenotype [133], further differentiation into PCs may increase XBP1s transactivation of *RTA*, leading to reactivation from latency and escape of the virus from a terminally-differentiated non-replicating host cell type. Conversely, KSHV may actively repress XBP1s activity to maintain tight control of latency, which may halt the differentiation process at a pre-plasma cell differentiation stage. PEL cells have also been shown to reactivate following prolonged ER stress through caspase-dependent cleavage of RAD21 [134]. RAD21 is a component of the cohesin complex that partners with CTCF to silence viral gene expression and maintain latency [135].

Control of MHV68 latent/lytic switch by XBP1s is only partially conserved; XBP1s transactivates the MHV68 *RTA* gene in vitro, but in vivo the plasma cell differentiation factor interferon regulatory factor 4 (IRF4) serves in this role [120]. The ER-localized MHV68 M1 protein is induced by RTA and IRF4 and may also play a role in reactivation from latency [136,137]. M1 also induces low levels of *XBP1* splicing [138], and may promote a feed-forward response to reactivate MHV68 by increasing XBP1s levels. M1 also upregulates ATF6-dependent ER chaperones BiP and Grp94, while preventing PERK phosphorylation of eIF2α, which may assist viral glycoprotein synthesis and folding.

**Figure 3:**
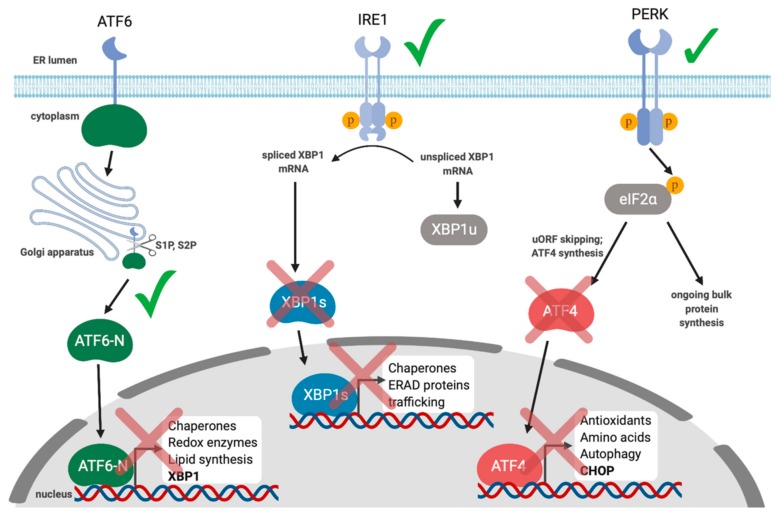
KSHV activates UPR sensors but limits UPR gene expression during the lytic cycle. XBP1s is required for reactivation from latency in response to ER stress. All three UPR sensor proteins are activated in the early stages of lytic replication, but downstream UPR transcription is inhibited. ATF6-N is produced via proteolytic cleavage in the Golgi, but ATF6-N-responsive genes are not transcribed. IRE1 is activated and *XBP1* is spliced, but XBP1s protein does not accumulate and XBP1s-responsive genes are not transcribed. eIF2α is phosphorylated in a PERK-dependent manner, but ATF4 protein does not accumulate and ATF4-responsive genes are not transcribed.

Collectively, these studies demonstrate a link between the UPR and gammaherpesvirus reactivation from latency and suggest that activation of XBP1s may be a conserved mechanism for reactivation. However, the subsequent interplay between these viruses and the UPR during lytic replication is less well understood. We recently demonstrated that lytic replication activates all three UPR sensors, which supports productive KSHV replication; inhibiting each UPR sensor via chemical inhibitors or RNA silencing diminished yield of infectious virions [139] (Figure 3). Despite clear UPR sensor activation, downstream UPR transcription was blunted in all three branches of the pathway. Specifically, we observed that PERK was activated during the lytic cycle and eIF2α was phosphorylated, but ATF4 did not accumulate, and accordingly, ATF4 target genes were not transcribed. Furthermore, ATF6 was proteolytically cleaved to release the ATF6-N bZIP transcription factor, but ATF6-N target genes were not transcribed. Finally, we observed that IRE1 was activated and *XBP1* mRNA was efficiently spliced during KSHV lytic replication, but XBP1s protein did not accumulate, and neither did products of XBP1s target genes. To determine whether UPR transcription might impact KSHV lytic replication, we ectopically expressed the spliced isoform of *XBP1*, which potently inhibited virion production in epithelial cells in a dose-dependent manner. This suggests that even though XBP1s plays an important role in reactivation from latency, the virus silences its expression during the lytic cycle to circumvent deleterious effects on viral replication. Currently we are unsure of how precisely XBP1s is blocking virus production, but it appears to be at a late step in the replication cycle.

There is supporting evidence that excessive UPR signaling inhibits KSHV lytic replication. Strong pharmacologic induction of the UPR with 2-deoxyglucose, brefeldin A, tunicamycin, or treatment with ER stress-inducing proteasome inhibitors (bortezomib, MG132, lactacystin, proteasome inhibitor I) can induce lytic reactivation while inhibiting virion production and triggering apoptosis [140,141,142,143]. We speculate that the virus may induce low levels of UPR activation to remodel the host cell and promote efficient lytic replication, but acute ER stress may still be detrimental to the virus due to the ensuing terminal pro-apoptotic UPR. This idea merits further investigation, but we should be cautious in interpreting studies that employ molecules that induce ER stress as a by-product of their primary mode of action.

It remains largely unclear how KSHV lytic replication activates UPR sensors while simultaneously inhibiting downstream UPR transcription. KSHV encodes the viral host shutoff RNA endonuclease SOX that targets the majority of host mRNAs for degradation [144,145]. As a result, this causes an indirect widespread transcriptional attenuation by repressing RNA polymerase II recruitment to host promoters [146]. We originally hypothesized that SOX-mediated repression of transcription during lytic replication was responsible for inhibiting the downstream transcriptional responses of the UPR; however, we showed that ectopic expression of SOX had no effect on UPR-responsive genes [139]. Having ruled out the most likely candidate UPR inhibitor, we speculate that the coordinated action of multiple viral gene products may be required to suppress UPR transcriptional responses.

The uORFs in *ATF4* mRNA are essential for ATF4 translation following eIF2α phosphorylation [34]. There are also uORFs in the KSHV genome, notably including ones that regulate the expression of the ORF35-ORF36-ORF37 locus [147,148]. It is not known how ISR activation impacts the translation of these uORF-containing viral mRNAs. KSHV may disrupt uORF-skipping and synthesis of host products like ATF4 as a by-product of controlling translation of viral uORF-containing mRNAs. XBP1s, ATF6-N, and ATF4 are bZIP transcription factors that can change the repertoire of genes that they regulate via heterodimerization [149]. KSHV encodes its own bZIP protein, K-bZIP, an RTA target gene that is expressed during the early stages of lytic replication [150]; it remains unknown whether K-bZIP suppresses the UPR by heterodimerization with host UPR transcription factors. K-bZIP also has small ubiquitin-like modifier (SUMO) ligase activity [151,152] that could influence the localization and/or fate of host UPR transcription factors. Notably, RTA itself has SUMO-targeted ubiquitin ligase (STUbL) activity [153] that could influence the fate of SUMOylated K-bZIP substrates. Thus, the failure of XBP1s or ATF4 proteins to accumulate during lytic replication may be due to SUMOylation and ubiquitination at the hands of viral proteins, followed by proteasomal degradation. Such studies could begin with a thorough analysis of the post-translational modifications and half-life of XBP1s during the lytic cycle.

One KSHV protein that could modulate UPR signaling during the lytic cycle is viral interleukin-6 (vIL-6; also called K2). vIL-6 has an amino-terminal signal peptide that directs its translation in the ER and subsequent secretion. However, a significant fraction of v-IL6 is retained in the ER where it interacts with hypoxia upregulated 1 (HYOU1, also called Grp170) [154]. HYOU1 is a nucleotide exchange factor that promotes ADP release from BiP, which allows for sustained BiP association with unfolded or misfolded proteins [155]. vIL-6 may interfere with HYOU1-BiP interactions, resulting in increased protein misfolding and UPR activation. However, there is also evidence that vIL-6 may promote protein folding by binding components of the calnexin cycle, including UDP-glucose: glycoprotein glucosyltransferase 1 (UGGT1) and glucosidase II (GlucII) [156]. Therefore, vIL-6 may play a role in fine-tuning UPR signaling by directly controlling proteostasis. Another study showed that there are XBP1s responsive elements in the vIL-6 promoter, suggesting that vIL-6 can accumulate in response to ER stress in a manner similar to RTA [157]. This arrangement could allow vIL-6 to quickly respond to changes in ER proteostasis.

In addition to vIL-6, products of the ORF47-ORF46-ORF45 locus may stimulate the UPR during the KSHV lytic cycle. P.-J. Chang et al. discovered new products of the tri-cistronic mRNA that encodes ORF47 (glycoprotein L), ORF46 (uracil DNA glycosylase), and ORF45 (multifunctional tegument protein), that activate the UPR [158]. In silico analysis of these alternatively spliced mRNAs suggested that they had the potential to encode two new variants of ORF45 dubbed ORF47/ORF45A and ORF47/ORF45B that contain 114 and 56 amino acids from the N-terminus of gL, respectively. Effectively, ORF47/ORF45A and ORF47/ORF45B are predicted to be fusions of the amino-terminal ER signal sequence of gL with the soluble tegument protein ORF45. Accordingly, ectopic expression of these two new gL/ORF45 fusion proteins revealed ER localization, whereas ORF45 remained in the nucleus and cytoplasm. Ectopic expression of ORF47/ORF45A and ORF47/ORF45B, but not ORF45, triggered *XBP1* splicing and BiP accumulation in HEK293T cells, whereas PERK and ATF6 were largely unaffected. It was proposed that the upregulation of these UPR markers was due to ER localization of these new ORF45 isoforms. Although the role that these alternative ORF45 isoforms play during lytic replication remains unclear, silencing BiP expression significantly reduced production of progeny virions, despite having little impact on viral gene expression. This suggests that BiP may be important for viral glycoprotein folding in the ER. However, BiP silencing also triggers ER stress and activates the UPR, so the diminished virion production may have been due to increased UPR signaling rather than viral protein folding activity of BiP. Therefore, further investigations into the effects of novel ORF45 protein isoforms on the UPR are warranted.

## 9. Cytomegalovirus (CMV) and the UPR

**Figure 4:**
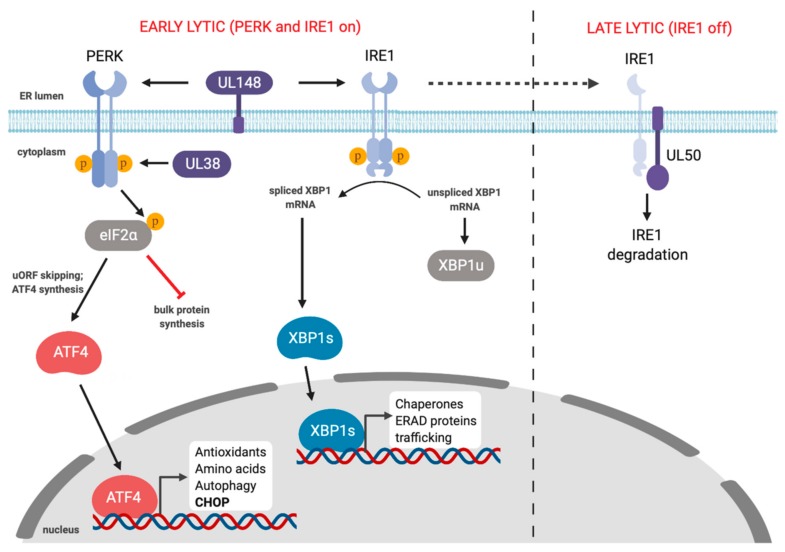
Differential control of the unfolded protein response by HCMV. In the early stages of HCMV lytic replication, PERK is activated by UL148 and UL38, which causes eIF2α phosphorylation and diminished global protein synthesis. At the same time, stress-dependent uORF skipping enables translation of the ATF4 bZIP transcription factor, which translocates to the nucleus and transactivates a variety of UPR genes. UL148 activates PERK as well as IRE1, which enables *XBP1* splicing and synthesis of the XBP1s bZIP transcription factor that transactivates a distinct set of UPR genes. Later in the lytic cycle, the UL50 protein binds and downregulates IRE1 through an unknown mechanism, effectively shutting down IRE1-dependent UPR gene expression.

Human CMV (HCMV) and murine CMV (MCMV) are betaherpesviruses that cause dramatic rearrangement of the cellular secretory pathway into a large perinuclear replication compartment that is visible by light microscopy [159,160,161,162]. Such dramatic changes in the ER compartment would be expected to impact ER proteostasis. Multiple studies have shown that HCMV and MCMV subvert the UPR during infection, likely as a means to support robust lytic replication. The first comprehensive study of UPR signaling in HCMV infection showed that the virus triggered IRE1-dependent splicing of *XBP1,* but the XBP1s gene target *EDEM* did not accumulate, suggesting a defect in downstream UPR transcription [163] (Figure 4). Likewise, HCMV infection activated PERK but elicited minimal eIF2α phosphorylation and bulk translation was largely unaffected; however, the ISR transcription factor ATF4 did accumulate, suggesting that HCMV exerts additional control over this arm of UPR. HCMV activation of PERK was also reported to promote virus replication through activation of SREBP1 by regulated intramembrane proteolysis, which then transactivates genes involved in lipid biosynthesis [164]. The mechanism of how HCMV infection triggers PERK-dependent SREBP1 cleavage is not known.

Multiple HCMV proteins influence UPR responses. The HCMV UL38 protein prolongs survival of infected cells and protects cells from ER stress-induced cell death by thapsigargin and tunicamycin [165,166]. UL38 is sufficient to promote cell survival by activating the PERK/eIF2α/ATF4 axis, while simultaneously suppressing pro-apoptotic IRE1-dependent JNK phosphorylation; ATF4 overexpression or JNK inhibition rescued cell viability following infection with UL38-deficient HCMV [167] (Figure 4). The ER-resident UL148 protein also activates PERK and promotes ATF4 accumulation [168]. Beyond PERK activation, UL148 also triggers IRE1 activation and *XBP1* splicing. UL148 remodels the ER and recruits components of the ERAD machinery to discrete compartments [169], most likely as a mechanism to increase the stability of viral glycoproteins like gO [170]. This ER remodeling by UL148 may activate the UPR by inhibiting proteasomal degradation of misfolded proteins. This ER remodeling is enhanced by PERK activation as treatment with ISRIB or a PERK inhibitor delayed the formation of UL148 foci [169]. ER remodeling also appears to be an evolutionarily distinct mechanism of HCMV UL148 to control ER reorganization and UPR activation, as the UL148 homologs from rhesus CMV and chimpanzee CMV do not perform this function [171].

Two additional HCMV proteins subvert ERAD as a mechanism to avoid immune detection. Unique short gene 2 and 11 (US2 and US11) directly interact with MHC-I proteins and target them for proteasomal degradation by using ERAD machinery [172,173,174,175]. At least for US11, XBP1 is required for robust ERAD of class I MHC heavy chain and was shown to stimulate low levels of XBP1 splicing and upregulate BiP, suggesting that US11 can likely induce the UPR [176]. Therefore, US11 is likely activating the UPR to ensure rapid degradation of MHC class I. Currently, it is not known if US2 and/or US11 coordinate with UL148 to re-direct ERAD components to ensure sufficient immune evasion through MHC-I degradation.

MCMV control of PERK is less nuanced, with PERK activation causing robust eIF2α phosphorylation, attenuation of bulk translation, and ATF4 accumulation, which aids virus production [177]. However, MCMV infection does not induce the accumulation of the ATF4 target protein CHOP, suggesting that the virus may intervene at this downstream step in the pathway.

MCMV and HCMV induce IRE1 activation and *XBP1* splicing early in infection, but at later stages of lytic replication IRE1 is downregulated, which impedes *XBP1* splicing (Figure 4). This block in *XBP1* splicing was also observed following pharmacologic induction of ER stress. IRE1 downregulation is mediated by the orthologous type II transmembrane MCMV M50 and HCMV UL50 proteins [178]. These proteins are already known to assemble with M53 and UL53, respectively, and accumulate in the inner nuclear membrane, where they act as a nuclear egress complex that helps newly packaged capsids breach the nuclear envelope and access the cytoplasm [179]. M50 and UL50 are sufficient for IRE1 downregulation without their NEC partner proteins, but the precise mechanisms of IRE1 suppression remain unknown. It is also not known how IRE1 downregulation might provide an advantage for HCMV or MCMV at late stages of infection. Release of MCMV progeny virions is diminished in XBP1-deficient cells [180], indicating that IRE1 activation early in the lytic cycle is likely required for efficient viral replication. Recently, Hinte et al. showed that early IRE1 activation following MCMV infection aids viral replication by depleting the cells of XBP1u [181]. They showed that *IRE1* knockout cells displayed reduced viral replication, which was restored in XBP1/IRE1 double knockout cells. XBP1u has a short half-life due to the presence of protein destabilizing domains in the C-terminus and can bind to XBP1s and ATF6 to promote proteasomal degradation [182,183]. Hinte et al. showed that XBP1s and ATF6 can bind to the MCMV major immediate early promoter (MIEP) and induce expression of the immediate early proteins IE1 and IE2 [181]. Therefore, activating IRE1 early in infection depletes the cell of XBP1u allowing XBP1s and ATF6 to bind the MIEP to facilitate early MCMV replication.

HCMV lytic replication selectively activates PERK and IRE1, but not ATF6 [163]. Nevertheless, the ATF6 target gene BiP is upregulated at the protein level in infected cells, likely through IRES-dependent translation [184]. BiP was also shown to colocalize with HCMV replication compartments, and decreasing BiP levels by RNA silencing or treatment with SubAB Shiga toxin disrupts formation of the replication complex, leading to a block in trafficking of viral nucleocapsids [185]. Therefore, it appears that HCMV upregulates BiP in an ATF6-independent manner. Collectively, these studies indicate that PERK activation is pro-viral for both HCMV and MCMV via multiple mechanisms, but roles for IRE1 and ATF6 are less well understood and may differ significantly between these viruses.

## 10. HSV-1 and the UPR

**Figure 5:**
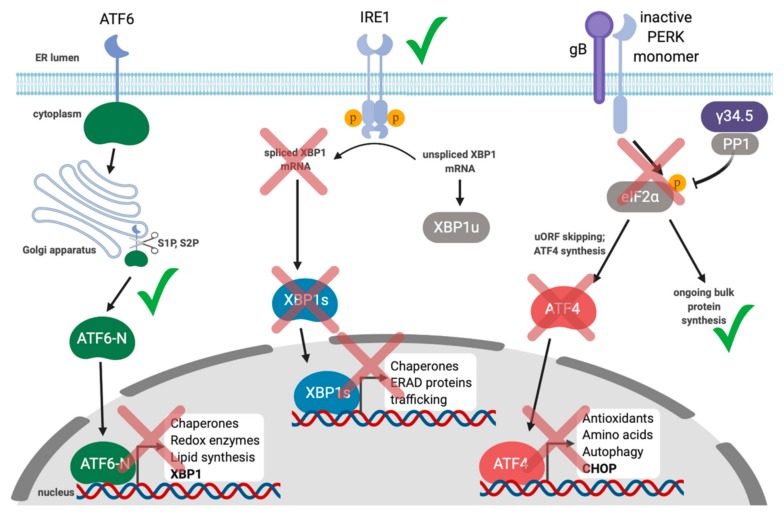
HSV-1 inhibits the UPR and the ISR. HSV-1 infection causes translocation of ATF6 to the Golgi apparatus, where is it cleaved by S1P and S2P to yield the ATF6-N bZIP transcription factor. Despite this, ATF6-N responsive genes are not transcribed in HSV-1 infected cells. HSV-1 triggers IRE1 kinase activity, but represses *XBP1* splicing and XBP1s synthesis, and subsequent XBP1s-dependent transcription. HSV-1 glycoprotein B (gB) binds to PERK and prevents its activation, thereby limiting eIF2α phosphorylation and maintaining bulk protein synthesis. HSV-1 γ34.5 protein acts similarly to GADD34, recruiting the cellular PP1 phosphatase to eIF2a and reinforcing the blockade on ISR activation. As a result, ATF4 is not synthesized and ATF4-responsive genes remain dormant.

HSV-1 is an alphaherpesvirus that also controls the UPR [186,187]. The best-known example of control over the UPR is that HSV-1 expresses an ortholog of GADD34 called γ34.5. Like GADD34, γ34.5 binds and recruits protein phosphatase 1 (PP1) to eIF2α, causing eIF2α dephosphorylation to ensure ongoing bulk protein synthesis even if eIF2α kinases like PKR and PERK become activated during viral infection [187,188] (Figure 5). HSV-1 Us11 protein can bind dsRNA and block PKR activation [189]. Ian Mohr’s group observed that a Us11 and γ34.5 double-knockout virus was still resistant to PERK activation in response to thapsigargin treatment, indicating that there may be other viral inhibitors of the UPR and/or the ISR that act directly on PERK [190]. They went on to show that the HSV-1 glycoprotein gB bound to PERK and this interaction was important for blocking eIF2α phosphorylation in response to stress, likely to enable viral protein translation to occur even in the presence of ER stress [191]. Thus, in contrast to CMV, PERK and ISR activation likely inhibits HSV-1 replication.

By maintaining a pool of unphosphorylated eIF2α, HSV-1 ensures translation of viral proteins while simultaneously preventing potentially detrimental downstream ISR transcription from ATF4 and CHOP, and induction of autophagy. Indeed, γ34.5 has been shown to antagonize autophagy by inhibiting the ISR as well as through direct binding to Beclin-1, a key autophagy protein [192,193]. By contrast, the alphaherpesvirus varicella zoster virus (VZV) may specifically induce the UPR as a mechanism to promote viral replication by upregulating autophagy [194,195].

HSV-1 replication has been shown to induce ATF6 cleavage but there is no impact on the ATF6 target gene BiP suggesting that HSV-1 blocks signaling of the ATF6 branch of the UPR [186]. IRE1 has also been reported to be activated during HSV-1 infection but its RNase activity is suppressed through an unknown mechanism [196]. Alternatively, IRE1 kinase activity and the IRE1-dependent phosphorylation of JNK was reported to promote virus replication, whereas XBP1s overexpression inhibits virus production. This suggests that HSV-1 fine-tunes IRE1 signaling to suppress XBP1s activation while activating the JNK pathway to promote HSV-1 replication [196]. Since XBP1s inhibits HSV-1, restoring IRE1 RNase activity may be one potential avenue of investigation for inhibiting HSV-1.

## 11. Conclusions and Outstanding Questions

The UPR is a conserved stress response that senses and responds to perturbations in ER proteostasis. Acute or chronic disruptions in ER protein folding can have drastic effects on the host if left unchecked. Therefore, in response to increases in ER protein misfolding the UPR transiently attenuates protein translation and selectively upregulates stress-responsive genes through the combined action of the transcription factors ATF4, XBP1 and ATF6. The end goal is to try to increase protein folding capabilities and degrade terminally misfolded proteins. Enveloped viruses may strain the protein folding machinery in the ER due to requirements for viral glycoprotein synthesis. As a result, these viruses likely have evolved mechanisms to ensure that different signaling responses of the UPR do not disrupt viral replication. Each branch of the UPR has a unique transcriptional output. Depending on the virus in question, the induced UPR genes could either be proviral or antiviral. Herpesviruses are large enveloped viruses and appear to have evolved to directly usurp UPR signaling, likely to dampen antiviral transcriptional responses while simultaneously promoting transcription of genes that promote virus replication. The pathways that are activated or inhibited greatly depend on the specific herpesvirus as well as the cell model.

UPR activation is the most reliable way to determine if a cell is experiencing ER stress. Concurrent activation of all three UPR sensors during infection suggests that the virus is inducing bona fide ER stress that causes BiP displacement from UPR sensor proteins. Alternatively, selective activation of UPR sensors could result from engagement with viral gene products in an ER stress-independent fashion. The development of new biochemical assays and reporter assays to detect ER protein misfolding will be helpful in differentiating between UPR-stimulating and UPR-inhibiting viral gene products. These studies will also be aided by the recent revolution in single-cell analysis of gene expression, which will provide richer information about the UPR transcriptional output during infection.

Potent ER stress-inducing drugs thapsigargin and tunicamycin have played a large role to date in UPR studies, but these drugs are often used at doses that do not recapitulate physiologic ER stress, and they also have known off-target effects. Thus, for studies that show modest activation of UPR sensors during virus infection and little UPR transcriptional output, it is unclear if UPR activation is too modest to elicit the anticipated transcriptional responses, or if the widespread use of thapsigargin and tunicamycin has left us with unreasonable expectations of transcriptional output. These studies will benefit from the development of more sensitive assays of UPR output and employing control molecules that more faithfully recapitulate the impact of infection stress on the ER.

As described above, there are links between UPR and innate and adaptive arms of the immune response. In this light, it is likely that herpesviral UPR modulation not only aids lytic viral replication but also aids viral evasion of immune surveillance. Improved understanding of the fundamental mechanistic links between the UPR and immunity should inform future studies of viral UPR subversion. Conversely, the study of UPR-modulating viral proteins could provide new tools to investigate functional interplay between the UPR and immunity.
